# Innovative Approaches in Dental Care: Electrical Impedance Analysis (EIA) for Early Caries Detection

**DOI:** 10.3390/bioengineering12030215

**Published:** 2025-02-20

**Authors:** Liliana Sachelarie, Ioana Romanul, Daniela Domocos, Mihaela Moisa, Emilia-Albinita Cuc, Raluca Iurcov, Carmen Stadoleanu, Loredana Liliana Hurjui

**Affiliations:** 1 Department of Preclinical Discipline, Faculty of Dental Medicine, Apollonia University, 700511 Iasi, Romania; drcarsta@yahoo.com; 2 Department of Dental Medicine, Faculty of Medicine and Pharmacy, University of Oradea, 10 1st Decembrie Street, 410073 Oradea, Romania; ioana_romanul@uoradea.ro (I.R.); ddomocos@uoradea.ro (D.D.); mmoisa@uoradea.ro (M.M.); cucalbinita@uoradea.ro (E.-A.C.); 3 Department of Morpho-Functional Sciences I, “Grigore T. Popa” University of Medicine and Pharmacy, University Street 16, 700115 Iasi, Romania; loredana.hurjui@umfiasi.ro

**Keywords:** electrical impedance analysis (EIA), dental microcracks, tooth structural fragility, non-invasive diagnostics

## Abstract

(1) Background: Microcracks and structural fragility in teeth, often undetected by traditional methods until severe complications like fractures or pulp exposure occur, are evaluated in this study using electrical impedance analysis (EIA) as a non-invasive tool for early detection and assessment. (2) Methods: A total of 57 patients were recruited, including individuals with bruxism (*n* = 20), dental restorations (*n* = 18), and no significant dental history (control group, *n* = 19). Electrical impedance measurements were performed on all teeth using a portable device, with data collected from occlusal and proximal surfaces. Patients with abnormal values underwent additional imaging (standard radiographs) to confirm the presence of microcracks. Statistical analyses included ANOVA to compare impedance values between groups and logistic regression to assess the predictors of structural fragility. (3) Results: Teeth with microcracks confirmed by standard radiographs exhibited significantly lower impedance values (mean 50 kΩ) compared to healthy teeth (mean 120 kΩ, *p* < 0.01). Patients with bruxism showed the highest proportion of teeth with abnormal impedance (45%). Logistic regression identified bruxism as a significant predictor of reduced impedance values (*p* < 0.05). (4) Conclusions: Electrical impedance analysis demonstrates promise as a non-invasive method for detecting microcracks and assessing structural fragility in teeth. Its application in routine dental check-ups could enable early interventions, particularly for high-risk patients with bruxism or restorations.

## 1. Introduction

Dental microcracks and structural defects are critical issues in dentistry, resulting in clinical complications such as fractured teeth and pulp exposure [1]. Traditional diagnostic methods, such as visual examination and radiographs, are not sensitive enough to detect such minute lesions, especially in patients with certain risk factors like bruxism, previously placed dental restorations, or occlusal irregularities [2,3].

Bruxism, characterized by clenching or grinding the teeth, applies excessive mechanical stress to tooth structures. Studies have shown that such repeated stresses can eventually result in enamel and dentin microcracks, increasing the predisposition to tooth fractures [4]. More recent data have established a correlation between nocturnal bruxism and enhanced tooth wear associated with the formation of microcracks, which tends to weaken mechanical teeth strength [5].

Dental cracks affect not only the structural integrity of the teeth but also the outcome of restorative treatments. Studies have shown that cracks can lead to restoration failure and secondary problems such as sensitivity or complete fractures [6,7,8].

In cases of cracks associated with bruxism, occlusal protective devices (mouth guards) can reduce mechanical stress, preventing the progression of cracks [4,6].

Finite element analysis studies have shown that crown design, occlusal contacts, and adhesive properties can influence the distribution of stresses in teeth. These stresses can promote the formation of cracks or contribute to their expansion [2,3]. Liu et al. have shown that malocclusion and cuspal inclination can affect the biomechanics of a premolar, increasing the likelihood of cracking in certain areas [3].

Dental restorations can also compromise tooth integrity. Though necessary to reinstate proper function, these procedures may stress tooth structures more, particularly in preparation modalities involving excessive hard tissue removals [6]. A study on the subject showed that using restorative materials with diverse biomechanical properties as alternatives could significantly change the pattern of the intratooth stress distribution [7].

Using restorative materials with different biomechanical properties can significantly alter how stresses are distributed in the tooth structure [3]. This approach is essential because each material, composite, ceramic, or metal alloy interacts differently with enamel and dentin. Materials with an elasticity close to that of natural tooth tissues can reduce the risk of microcracks as they distribute stresses more evenly, preventing them from being concentrated in specific points [4]. For example, although durable and aesthetic, ceramic restorations generate higher stresses at the interface between the restoration and the natural tooth, increasing the risk of subclinical cracks [8,9]. In contrast, composite materials can absorb some of the masticatory forces due to their elasticity, thus reducing stress on the tooth structure. Özcelik et al. (2011) highlighted that using non-rigid connectors in dental restorations can help to reduce stress concentrations, protecting the tooth’s structural integrity [4].

These findings highlight the importance of customizing restorative treatments based on each patient’s characteristics and the biomechanical needs of the affected tooth. In addition, pre- and post-restoration assessments using advanced methods such as electrical impedance analysis or standard radiograph imaging can identify risk areas and optimize the restorative treatment’s longevity [10,11].

Standard diagnostic methods, including visual and radiographic examinations, have significant limitations regarding detecting such lesions in their early stage. Two-dimensional X-rays cannot disclose tiny or poorly oriented cracks, while visual examinations are subjective and highly dependent on the clinician’s experience [8]. Along these lines, novel technologies such as standard radiographs and multi-modal imaging analyses have the potential to provide improved accuracy in dental microcrack detection; however, they are, in turn, limited by high costs and availability issues [9,10].

Standard radiographs and other advanced imaging techniques, such as contrast-enhanced computed tomography or near-infrared (NIR) imaging, have been used to assess microcracks in the enamel and dentin structure. These methods provide detailed visualizations of the location and depth of cracks [7,10].

Contrast-enhanced cone beam computed tomography (CBCT) is a recently introduced technique that improves the detection of microcracks and provides precise information about their depth [10].

Dumbryte used standard radiographs to visualize microcracks in the dental enamel in three dimensions. The study highlighted the role of noninvasive technologies in assessing dental damage and preventing the loss of dental function [7].

Zhou and his team introduced a contrast-enhanced CBCT-based method (cone beam computed tomography) for crack detection in vitro, demonstrating increased sensitivity compared to traditional radiography [10].

Emerging evidence suggests that noninvasive EIA (electrical impedance analysis) may be a promising method for detecting microcracks and assessing tooth fragility. EIA measures the electrical resistance of dental tissue, which reflects changes in structure, such as demineralization or cracks [11]. Initial reports suggest that EIA might be a valuable tool in the early identification of high-risk areas and, thus, might help to avoid complications [12,13].

Electrical impedance analysis (EIA) is a modern and non-invasive diagnostic method that measures the resistance of dental tissues to the passage of a weak electric current. The basic principle of this method is that the structure and composition of dental tissue influence its electrical properties. When changes occur, such as microcracks or demineralization, the impedance values change, which can be detected using this technique. Studies have shown that such impedance changes indicate early structural damage in dental tissues [3,4].

EIA allows for the identification of incipient structural changes in the enamel and dentin, which are difficult to observe with the naked eye or through traditional radiographs. For example, Liu et al. (2014) highlighted how subtle biomechanical changes in the tooth structure, such as cusp inclination or occlusal stress, influence dental integrity. They can be effectively assessed through advanced diagnostic tools [3]. This method can detect areas with a high risk of cracking or demineralization early, providing the opportunity to intervene preventively [5].

Unlike radiographs or other imaging methods, EIA does not involve radiation exposure, can detect subtle changes that may go unnoticed by traditional methods, and is rapid in its measurements. Devices used for EIA are portable and easy to use, making them suitable for routine dental practice and preventive care [6].

This research aims to investigate the feasibility of EIA in determining dental microcracks at an early stage and formulating strict criteria to differentiate between healthy and structurally weak teeth. Incorporating this technology will improve prevention and treatment options in dental practice.

## 2. Materials and Methods

### 2.1. Study Design

This prospective and observational study focused on assessing dental microcracks and structural fragility using electrical impedance analysis (EIA) as a noninvasive method. The aim was to detect early microcracks and evaluate the specific predictors of dental structural fragility.

### 2.2. Participants

The study included a total of 57 patients aged between 25 and 60 years who were recruited from Apollonia Dental Clinic based on the following inclusion criteria: Group 1: patients diagnosed with bruxism (*n* = 20) based on their history and clinical signs of dental abrasion; Group 2: patients with extensive dental restorations (*n* = 18), including multiple crowns and fillings; and Group 3 (control): patients without a significant dental history or dental problems (*n* = 19).

This study received approval from the Ethics Committee of Apollonia University under reference number 59, granted on 28 November 2023. Ethical clearance was provided in accordance with institutional and international guidelines, including the Declaration of Helsinki. All participants provided written informed consent before enrollment.

### 2.3. Measurement Methodology

Electrical impedance measurements were performed using a CarieScan Pro device (CarieScan Ltd., Scotland, Dundee, UK). The CarieScan Pro is a portable impedance analysis device specifically designed for dental diagnostics. Before each session, the device was calibrated using a standard reference solution to ensure consistent and accurate results. Disposable electrodes were used for each measurement, ensuring hygienic application, and the device’s intuitive software interface allowed for real-time data acquisition and analysis.

Before the measurements, all teeth were carefully cleaned and dried to eliminate saliva or other residue that could interfere with the impedance readings. The probe’s active electrode was applied perpendicularly to each tooth’s occlusal and proximal surfaces, while the reference electrode completed the electrical circuit. Care was taken to avoid contact with the gingiva, ensuring that only the dental tissues influenced the measurements. Each reading lasted approximately three seconds per surface, with impedance values recorded automatically in kilohms (kΩ) and transferred to the dedicated software for analysis.

Teeth with impedance values below the threshold (<60 kΩ) were flagged for further investigation. For these cases, high-resolution micro-computed tomography (micro-CT) imaging was performed to confirm the presence and extent of microcracks or other structural anomalies. Standard radiographs provided a detailed three-dimensional visualization, ensuring that abnormalities detected by EIA corresponded to actual defects in the dental structure.

The measurement process was highly standardized. To maintain reliability, all measurements were conducted by the same operator under consistent environmental conditions.

The device was recalibrated regularly between sessions to ensure accuracy, and the collected data were systematically analyzed to differentiate between normal and abnormal structural patterns.

Combining the rapid, non-invasive assessment of EIA with the detailed imaging capabilities of micro-CT, this methodology provided a robust and precise approach to detecting microcracks and assessing structural fragility in teeth.

### 2.4. Statistical Analyses

The impedance values between the three groups were analyzed using a one-way analysis of variance (ANOVA). Post hoc comparisons were performed using the Tukey Honest Significant Difference (HSD) test to identify specific differences between group means. Logistic regression was used to evaluate predictive structural fragility factors, such as bruxism or dental restorations. The significance threshold was set at *p* < 0.05.

The formula for calculating electrical impedance Z is as follows:

$$Z=\frac{V}{I}$$

*Z*: the electrical impedance (in ohms, Ω);

V: the applied voltage (in volts, V);

I: the measured current (in amperes, A).

The mathematical model used for the ANOVA analysis is expressed as

$$Y_{ij}=\mu +\alpha_{i}+\varepsilon_{ij}$$

$Y_{ij}:$ the observed impedance value for subject *j* in group *i*;

μ: overall mean;

$\alpha_{i}$: effect of group I;

$\varepsilon_{ij}$: residual error associated with observation $Y_{ij}$.

In this study, the groups analyzed were the control group, Group A, and Group B, each containing 15 patients. The model was applied to evaluate significant differences between the electrical impedance means of these groups, considering the internal variations specific to each group.

To identify the significant differences between the analyzed groups (control group, Group A, and Group B), the Tukey Honest Significant Difference (HSD) test was used, expressed by the formula

$$q=\frac{\bar{Y_{I}}-\bar{Y_{J}}}{SE}$$

In this formula, $\bar{Y_{I}}-\bar{Y_{J}}\;are\;the\;mean\;groups\;i\;and\;j$ and SE is standard error.

The test allowed for a post hoc comparison between all groups, highlighting the statistically significant differences between the mean electrical impedance values.

## 3. Results

### 3.1. Baseline Characteristics

Table 1 presents the basic characteristics of the three groups of patients included in the study, highlighting the relevant differences according to their age, gender, average number of teeth, and the specific distribution according to the risk factors studied (bruxism, dental restorations, and control group). The group of patients with dental restorations (Group 2) has a higher mean age (40.3 ± 6.2 years) than the other groups. This may reflect that dental restorations become more common with advancing age. The patients in the control group and those with bruxism have a similar mean age (37.5 ± 4.9 and 35.2 ± 5.8 years, respectively), which ensures a balanced comparison between the groups. The ratio of men to women is relatively balanced in all groups, without major differences: Group 1 (bruxism) has twelve men and eight women; Group 2 (restorations) has nine men and nine women; and Group 3 (control) has ten men and nine women.

The control group has the highest mean number of teeth (30 ± 1), indicating good oral health. Patients with bruxism have a slightly lower mean (28 ± 3), possibly due to abrasion or fractures caused by teeth grinding. The group with dental restorations has the lowest mean number of teeth (26 ± 2), suggesting significant tooth loss or previous treatments.

All patients in Group 1 (100%) have this condition, confirming targeted selection. Group 2 comprises patients with dental restorations (100%), ensuring a clear comparison between the categories. Group 3 includes patients without relevant dental problems (100%), providing an essential reference point for assessing standard impedance.

### 3.2. Electrical Impedance Measurements

Electrical impedance measurements highlight significant differences in the mean electrical impedance values and the distribution of teeth with low impedance values (<60 kΩ) between the three studied groups, as seen in Table 2. The mean electrical impedance values are the lowest (50 ± 5 kΩ), indicating significant structural fragility in teeth affected by this condition. The proportion of teeth with abnormal values is the highest of the three groups (45%), confirming the impact of bruxism on microcracks and structural fragility. This can be explained by the excessive mechanical forces exerted on enamel and dentin during teeth grinding.

The restored teeth present mean impedance values higher than those in the bruxism group (60 ± 8 kΩ), but still below the normal threshold for healthy teeth. The proportion of teeth with low impedance values is 32%, reflecting residual stresses and possible cracks associated with dental restorations. This suggests that extensive restorations may weaken the tooth structure, especially at the restoration–dental tissue interface. Healthy teeth in the control group have the highest mean impedance values (120 ± 10 kΩ), reflecting their good structural integrity. The proportion of teeth with low impedance values is minimal (5%), indicating a low prevalence of microcracks in this group. These data serve as a reference point for comparison with the affected groups.

Table 3 presents a comparative analysis of the mean impedance values (in kΩ) among the three study groups, highlighting significant differences in structural integrity as measured by electrical impedance analysis (EIA).

The mean impedance value for Group 1 is the lowest, at 50 ± 5 kΩ. This indicates substantial structural fragility and the presence of microcracks, likely caused by the repetitive mechanical forces exerted during teeth grinding.

The F-statistic of 12.8 and the *p*-value < 0.01 confirm that this group’s mean impedance value is significantly different from the other groups, reflecting the strong impact of bruxism on dental structure.

The mean impedance value for Group 2 is slightly higher than the Bruxism group, at 60 ± 8 kΩ, but still well below the normal range. This suggests that the stresses introduced by dental restorations, such as tension at the restoration–tooth interface, contribute to structural compromise. The mean impedance value for Group 3 is the highest, at 120 ± 10 kΩ, indicating intact dental structures with minimal microcracks or fragility. The control group serves as the baseline, and its significantly higher impedance values highlight the normal structural integrity of healthy teeth.

The F-statistic value of 12.8 and the highly significant *p*-value (<0.01) confirm that the differences in mean impedance values between groups are statistically significant.

Table 4 summarizes the logistic regression analysis results used to identify the significant predictors of low impedance values (indicating dental fragility or microcracks). The analysis focuses on bruxism and restorations.

The OR for bruxism is 3.8, indicating that patients are approximately 3.8 times more likely to exhibit teeth with low impedance values than those without bruxism. This finding underscores the significant impact of bruxism on dental structural integrity, likely due to repetitive mechanical stress and wear caused by teeth grinding or clenching.

The OR for restorations is 2.4, suggesting that patients with extensive dental restorations are 2.4 times more likely to have teeth with low impedance values than those without restorations. The associated *p*-value (<0.05) confirms that this result is statistically significant, indicating that restorations contribute meaningfully to structural changes in the tooth, possibly due to stress at the restoration–tooth interface.

**Figure 1:**
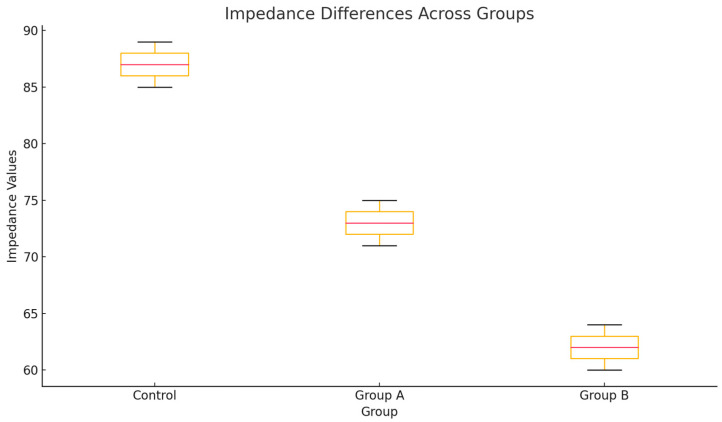
Electrical impedance differences between groups.

Figure 1 highlights the differences in the electrical impedance values between the analyzed groups (control, Group A, and Group B). The control group presents higher impedance values, while Groups A and B have lower values, indicating greater structural fragility. The differences between the groups are statistically significant according to ANOVA analysis and Tukey’s test, suggesting an essential variation in the microstructural state between these categories.

Tukey’s HSD test results indicate statistically significant differences between all pairs of groups analyzed. The control group presents higher electrical impedance values than Groups A and B, suggesting the better structural integrity of the teeth in this group. Also, Group B presents the lowest impedance values, highlighting a greater structural fragility than Group A. These differences highlight the significant variations in the dental microstructure between groups, confirming the relevance of the analysis performed.

Standard radiographs were used to confirm the presence of microcracks in teeth with abnormal impedance values, as seen in Table 5.

In the bruxism group, 42% of the teeth had confirmed microcracks, reflecting the direct impact of repetitive mechanical forces on enamel and dentin.

In the restoration group, 28% of the teeth had confirmed microcracks. These may be caused by stresses induced during the preparation and application of dental restorations.

Only 4% of the teeth in the control group had microcracks, confirming that increased electrical impedance indicates normal structural integrity.

## 4. Discussion

This study demonstrated significant differences in the mean impedance values between patient groups and correlated these values with the presence of dental microcracks, confirmed by micro-CT. The group of patients with bruxism had the lowest impedance values and the highest prevalence of microcracks (42%), supported by the literature. For example, Yap et al. (2023) highlighted that microcracks frequently occur in cases of bruxism due to the repetitive mechanical stress that compromises the enamel structure [1].

Also, the group with dental restorations showed an increased prevalence of microcracks (28%), which are associated with stresses induced by restorative materials. These observations align with the analysis made by Shahrbaf et al. (2013), who demonstrated that the stresses generated at the interface between the restoration and the tooth can induce microcracks, especially in the case of prosthetic crowns [2].

These findings validate the sensitivity of electrical impedance analysis as a tool for distinguishing between healthy teeth and those affected by conditions such as bruxism or the presence of restorations—the utility of mean impedance values as a reliable metric for assessing dental structural integrity. The data emphasize that teeth affected by bruxism or restorations exhibit significantly lower impedance values than healthy teeth, underscoring the potential of EIA in the early detection and prevention of structural dental issues.

The control group having a minimal prevalence of microcracks (4%) validates high electrical impedance values as an indicator of normal dental health.

The presence of microcracks in the group of patients with bruxism (42%) is aligned with the studies of Kang et al. (2016), who showed that teeth grinding is a significant risk factor for the formation of dental cracks, with a reduced survival rate of these teeth after treatment [12]. On the other hand, in the group with dental restorations, the prevalence of micro-CT-confirmed microcracks (28%) is similar to the observations made by Rivera and Walton (2015), who highlighted that those restorative interventions can weaken the tooth structure by introducing internal stresses [7,13,14].

The higher OR for bruxism than restorations suggests that bruxism is a stronger predictor of low impedance values and structural fragility. The statistically significant results for restorations (*p* < 0.05) emphasize the potential long-term impact of restorative procedures on dental health.

Extensive dental restorations are recognized as a significant risk factor for microcracks and structural fragility. Lubisich et al. (2010) highlighted that cracks can occur around restorations, especially in the case of invasive techniques such as full crowns or extensive fillings, which involve the significant removal of tooth tissue [15]. These observations are consistent with the results of our study, which identified low impedance values (60 ± 8 kΩ) in the restoration group, reflecting structural changes caused by residual stresses.

Härle et al. (2003) analyzed the differences between ceramic and composite restorations regarding stress distribution and crown flexure. Their study showed that ceramic materials generate higher stresses at the enamel interface, contributing to subclinical cracks [16,17,18,19,20,21,22,23,24,25]. These findings suggest that the selection of restorative materials should consider the impact on structural integrity, a recommendation supported by the logistic analysis in this study (OR = 2.4 for restorations).

Cameron introduced the concept of “cracked tooth syndrome”, highlighting the subtle clinical signs that may precede overt fractures [18,19,20,21,22,23,24,25,26,27,28]. Subsequent studies, including Kang et al. (2016), have confirmed that teeth grinding increases the risk of cracking, and ill-fitting dental restorations can amplify these effects [12]. This explains why patients with bruxism had the highest proportion of teeth with microcracks (42%) in this study.

Noninvasive imaging plays a central role in the early detection of microcracks. Dumbryte et al. (2021) demonstrated that standard radiographs can visualize enamel microcracks in detail, and three-dimensional analysis helps to quantify stresses in the tooth structure [29]. Similarly, Zhou et al. (2022) used contrast-enhanced CBCT to assess crack depth, which complements this study’s electrical impedance screening method [19].

Bishara et al. (2008) and Dumbryte et al. (2016) investigated the effects of orthodontic procedures on enamel. They found that the debonding and adhesive removal process contributes to the formation of microcracks [20,21]. These findings suggest that patients with a history of orthodontic treatment may benefit from periodic structural integrity assessments using methods such as electrical impedance.

This analysis highlights the importance of monitoring patients with bruxism and extensive restorations, as both conditions significantly increase the likelihood of structural dental compromise. Logistic regression reinforces the relevance of these risk factors in predicting low impedance values, further supporting the role of electrical impedance analysis as a diagnostic tool in high-risk populations.

The use of standard radiographs in this study allowed for the accurate detection of microcracks and confirmed the reliability of electrical impedance as a screening method. Similarly, Zhou et al. (2022) demonstrated the effectiveness of advanced techniques, such as contrast-enhanced CBCT, in assessing the depth of dental cracks [19]. Also, Dumbryte et al. (2021) highlighted the importance of standard radiographs in the three-dimensional visualization of enamel cracks, supporting the utility of this technology in dental diagnosis [29]. Electrical impedance analysis, in combination with standard radiography imaging, provides a practical and sensitive method for detecting microcracks and assessing structural fragility.

The electrical impedance analysis is a robust method for detecting dental microcracks. Factors such as bruxism and extensive dental restorations significantly influence structural fragility. The results support integrating this technology into preventive dental practice, especially for high-risk patients.

Logistic regression indicated that bruxism was the strongest predictor of low impedance values (OR = 3.8), which is in line with the findings of Vladutu et al. (2022), who described bruxism as a disorder characterized by excessive mechanical stress, with direct implications for dental micro fissures [6]. Dental restorations were also a significant predictor (OR = 2.4), highlighting the need for minimally invasive restorative strategies, as recommended by Lubisich et al. (2010) [15].

Dumbryte et al. (2017) showed that visible microcracks are correlated with increased tooth sensitivity, a common symptom reported by patients with bruxism or extensive restorations [23,24]. This association supports using electrical impedance analysis as a screening tool, as low values may indicate areas prone to sensitivity or cracking.

In recent studies, emerging technologies, such as near-infrared fluorescence (ICG-NIR) and laser scanning, have been used for crack detection. Sun et al. (2014) demonstrated the effectiveness of laser scanning in identifying cracks at early stages [24], while Li et al. (2020) showed that NIR fluorescence can provide detailed information about crack depth [25,26,27,28,29]. These techniques can complement electrical impedance-based methods for accurate diagnosis.

The meta-analysis studies by Dumbryte et al. (2018) emphasized the need to prevent dental fissures by reducing risk factors, such as bruxism, and using dental materials that distribute stresses evenly [30,31]. This study’s results support these conclusions, highlighting bruxism as the main predictor of dental fissures (OR = 3.8).

The Tukey test confirms that all differences in means between the analyzed groups (control, Group A, Group B) are statistically significant. This highlights the important variations in electrical impedance between the groups, which are associated with differences in the integrity and fragility of the dental microstructure.

The results of this study support the integration of electrical impedance analysis as an early diagnostic method for dental microcracks, complemented by standard radiograph imaging for high-risk cases. In addition, prevention strategies, such as occlusal protection for patients with bruxism and using restorative materials that reduce internal stresses, could reduce the prevalence of microcracks. Our study highlights that electrical impedance analysis and advanced imaging can detect early microcracks and dental fragility. Integrating these methods into clinical practice could prevent complications associated with bruxism, dental restorations, and orthodontic treatments.

## 5. Conclusions

Through this study, we have shown that electrical impedance analysis (EIA) is effective in the early detection of dental microcracks and structural fragility, with the results being correlated with bruxism and extensive restorations. Integrating EIA into dental practice could improve the prevention and monitoring of high-risk patients, complementing advanced imaging methods for more accurate diagnosis.

## Figures and Tables

**Table 1:** Baseline characteristics of groups.

Characteristic	Group 1 (Bruxism)	Group 2 (Restorations)	Group 3 (Control)
Age (mean ± SD)	35.2 ± 5.8	40.3 ± 6.2	37.5 ± 4.9
Gender (M/F)	12/8	9/9	10/9
Number of teeth (mean ± SD)	28 ± 3	26 ± 2	30 ± 1
Bruxism (%)	100	0	0
Restorations (%)	0	100	0

**Table 2:** Electrical impedance measurements.

Group	Mean Impedance (kΩ ± SD)	Teeth with Low Impedance (%)
Bruxism	50 ± 5	45% (45/100)
Restorations	60 ± 8	32% (36/113)
Control	120 ± 10	5% (6/120)

**Table 3:** Comparison of mean impedance values between groups.

Group	Mean Impedance (kΩ ± SD)	F-Statistic	*p*-Value
Bruxism	50 ± 5	12.8	<0.01
Restorations	60 ± 8	-	-
Control	120 ± 10		

**Table 4:** Logistic regression—predictors of low impedance values.

Predictor	Odds Ratio (OR)	*p*-Value
Bruxism	3.8	<0.05
Restorations	2.4	<0.05

**Table 5:** Standard radiograph imaging—presence of confirmed microcracks.

Group	Teeth with Microcracks Confirmed by Standard Radiographs (%)
Bruxism	42%
Restorations	28%
Control	4%

## Data Availability

The data results can be found at corresponding author.
